# Predicting proximal junctional failure in adult spinal deformity patients using machine learning models based on spinal alignment parameters

**DOI:** 10.1038/s41598-025-24872-1

**Published:** 2025-11-20

**Authors:** Akihiko Hiyama, Daisuke Sakai, Hiroyuki Katoh, Masato Sato, Masahiko Watanabe

**Affiliations:** Department of Orthopaedic Surgery, Surgical Science, Tokai University School of Medicine, 143 Shimokasuya, Isehara, Kanagawa 259-1193 Japan

**Keywords:** Diseases, Health care, Neurology

## Abstract

Proximal junctional failure (PJF) is a significant mechanical complication following corrective surgery for adult spinal deformity (ASD), often resulting in structural failure at the uppermost instrumented vertebra and necessitating revision surgery. Early identification of patients at high risk for PJF remains clinically important but challenging due to the multifactorial and nonlinear nature of risk factors. This study aimed to evaluate the predictive performance of five machine learning (ML) models—Random Forest, Logistic Regression, Support Vector Machine (SVM), Decision Tree, and Naive Bayes—in identifying patients at risk for PJF using both preoperative and postoperative spinal alignment parameters. A retrospective cohort of 92 ASD patients who underwent two-stage corrective surgery, including lateral lumbar interbody fusion (LLIF), was analyzed. Radiographic parameters were measured preoperatively and approximately six weeks postoperatively. Six alignment-related features were selected through a combination of univariate statistical testing and Random Forest–based feature importance ranking: preoperative PI–LL, preoperative PT, postoperative PI–LL, postoperative and preoperative TK, and change in lumbar lordosis (ΔLL). Each ML model was trained and tested using five independent stratified 80:20 train-test splits. Among the models, Random Forest achieved the highest mean accuracy (78.4%) and area under the curve (AUC = 0.704). The predicted probabilities for PJF were significantly higher in the PJF group compared to non-PJF cases (0.306 ± 0.181 vs. 0.186 ± 0.164, *p* = 0.0057). Cross-validation confirmed model robustness (fivefold: 79.4%, tenfold: 77.3%). These findings suggest that Random Forest can serve as a reliable tool for preoperative and early postoperative PJF risk stratification based on alignment correction. Future work should incorporate bone mineral density, comorbidities, and multicenter validation to enhance clinical applicability.

**Supplementary Information:**

The online version contains supplementary material available at 10.1038/s41598-025-24872-1.

## Introduction

Proximal junctional failure (PJF) is a known complication following spinal fusion surgery, often necessitating additional interventions due to adverse outcomes^1,2^. PJF represents a structural failure at spinal segments adjacent to the fusion, presenting as vertebral fractures, ligament injuries, or deformities in the proximal junctional area^3^. This issue is particularly significant in patients undergoing corrective surgery for adult spinal deformity (ASD), where it threatens the durability and success of surgical interventions.

Achieving optimal lumbar lordosis (LL) is crucial in ASD correction, as inadequate spinal alignment is associated with increased risks of complications such as PJF. To guide spinal alignment, various formulas have been proposed, including PI-LL ≤ 10°,^4^ LL + PI + TK ≤ 45°,^5^ and other equations based on pelvic incidence (PI) and patient age, such as ideal LL = 0.45 × PI + 31.8,^6^ ideal LL = 0.59 × PI + 11.1,^7^ and ideal LL = 0.6 × PI + 32.9–0.23 × age^8^. Although these formulas provide useful corrective targets, they cannot fully predict the occurrence of PJF in every case, as these complications are influenced by multiple factors that a linear model may not capture.

The computerized decision support tool initially developed for ASD had certain limitations, with predictive models at the time achieving only around 70% accuracy^9–11^. This limitation highlighted the need for further improvements, as increasing the number of observed variables alone did not enhance predictive precision. Instead, introducing new “dimensions” or data points was required to better capture the complexity of PJF risk factors. However, recent advancements in machine learning have led to more sophisticated models capable of handling multidimensional and nonlinear relationships more effectively.

In this study, we evaluated five machine learning (ML) models—Random Forest, Logistic Regression, Support Vector Machine (SVM), Decision Tree, and Naive Bayes—to predict the occurrence of PJF. Each model was selected for its unique strengths in analyzing nonlinear and multifactorial relationships^12–14^: Random Forest can effectively capture feature importance and handle complex feature interactions; Logistic Regression provides interpretable coefficients for linear relationships; SVM excels in handling high-dimensional and nonlinear data with its kernel trick; Decision Tree offers intuitive, rule-based classification; and Naive Bayes is computationally efficient, particularly for high-dimensional data. These qualities may make them suitable for exploring the diverse clinical and alignment factors contributing to PJF.

This study aims to advance the field by applying machine learning models to predict PJF occurrence based on both preoperative and postoperative spinal alignment parameters, addressing the limitations of earlier predictive models. Unlike prior research on linear models with limited variables, we incorporate differential data capturing changes between preoperative and postoperative measurements. By incorporating these changes over time, our approach enhances the model’s complexity and clinical applicability, potentially providing a more accurate means of identifying high-risk patients for PJF.

By enabling early identification of high-risk patients for PJF, these models have the potential to support clinicians in making data-driven decisions regarding postoperative management and preventive strategies. For instance, targeted interventions and more intensive postoperative monitoring might be provided to those identified as high-risk, potentially improving surgical outcomes and reducing reoperation rates.

## Material and methods

### Ethics approval

The study protocol was reviewed and approved by the Ethics Committee, Institutional Review Board, and the Profit Reciprocity Committee of Tokai University School of Medicine. Due to the retrospective nature of the study, the Research Ethics Committee of Tokai University School of Medicine waived the need to obtain informed consent　(Approval No. 24R-143).

All methods were carried out in accordance with relevant guidelines and regulations.

### Study design and data collection

Between January 2016 and April 2024, a total of 106 ASD patients underwent two-stage corrective surgery due to poor spinal alignment.

The standard two-stage anterior–posterior approach for correcting degenerative scoliosis was performed as follows: First, lateral lumbar interbody fusion (LLIF) was performed via a lateral approach at 2–4 intervertebral levels. Large cages (8–12 mm in height, 10° angle) were inserted to correct and stabilize the intervertebral bodies. Patients were encouraged to ambulate the day after surgery, and spinal alignment was reassessed to plan the second-stage procedure. One week later, posterior corrective fusion was performed using a pedicle screw system, with transforaminal lumbar interbody fusion (TLIF) at L5/S1 added for further stabilization^15^.

Inclusion criteria for this study included a minimum follow-up period of one year, fusion of at least six vertebrae, and the lowest instrumented vertebra being at the sacrum. Additionally, radiographic inclusion criteria for ASD included at least one of the following: coronal Cobb angle ≧ 20°, sagittal vertical axis (SVA) ≧ 5 cm, pelvic tilt (PT) ≧ 25°, and thoracic kyphosis (TK) ≧ 60°^15–19^. Exclusion criteria included incomplete radiographic or clinical follow-up, prior fusion surgery history, and circumferential fusion absence.

All patients underwent full-length anteroposterior and lateral standing X-rays, assessed for coronal alignment and sagittal spinopelvic parameters. Patient demographic and clinical data, including age, sex, and preoperative and postoperative spinal alignment parameters (SVA, PT, TK, pelvic incidence (PI), sacral slope (SS), lumbar lordosis (LL)), were collected. This retrospective study ultimately included data from 92 adult patients (aged ≥ 20) who met the above criteria and underwent two-stage corrective surgery for ASD, with LLIF as part of the procedure.

Postoperative spinal alignment parameters were evaluated at an average of 6.3 ± 6.5 weeks following surgery. The differential data for these parameters (changes from preoperative to postoperative values) were calculated to assess their impact on the risk of PJF. Among the 92 patients, 21 (22.8%) developed PJF, as defined in our study. PJF is an advanced form of proximal junctional kyphosis, characterized by vertebral fracture or subluxation, failure at the UIV or UIV + 1 level, implant loosening, or neurological deficits, which often necessitate reoperation due to clinical symptoms^20^. In this study, we classified PJF cases only when these complications required reoperation. We have defined PJF in accordance with this concept. The average follow-up period was 25.5 ± 14.9 months. Although the minimum follow-up was set at 1 year to exclude early dropouts, patients who developed PJF beyond the first postoperative year were also included as cases of PJF. Thus, our labeling of outcomes reflects the occurrence of PJF events throughout the entire available follow-up period.

Surgical details, such as the number of fused vertebrae, LLIF segments, operation times for LLIF and PSF, and intraoperative estimated blood loss, were documented to provide a comprehensive patient profile. Patient demographic data are shown in Table 1.

**Table 1:** Demographics of all patients. Data presented as mean (SD) or number of patients (%). HT, height; BW, body weight; BMI, body mass index; LLIF, lateral lumbar interbody fusion; PSF, posterior spine fusion; EBL, estimated blood loss; SVA, sagittal vertical axis; PT, pelvic tilt; PI, pelvic incidence; SS, sacral slope; LL, lumbar lordosis; TK, thoracic kyphosis; UIV, upper instrumented vertebra. Δ indicates the difference calculated by subtracting the postoperative and preoperative measurements.

Variable		ALL
N		92
Age (yrs)		71.1 (8.4)
60y <		82 (89)
Woman, n		83 (90)
Follow-up periods(M)		25.5 (14.9)
HT (cm)		150.3 (7.7)
BW (kg)		51.0 (10.2)
BMI (kg/m^2^)		22.6 (3.8)
SVA	Pre	160.0 (71.0)
	Post	28.0 (48.0)
	Δ	− 132.0(76.7)
PI	Pre	49.8 (9.1)
	Post	46.6 (8.7)
	Δ	− 3.2 (6.7)
PT	Pre	33.7 (9.5)
	Post	15.9 (8.9)
	Δ	− 17.9 (9.4)
SS	Pre	16.1 (9.4)
	Post	30.9 (8.9)
	Δ	14.8 (9.3)
LL	Pre	6.7 (22.4)
	Post	50.9 (13.2)
	Δ	44.1 (21.5)
PI-LL	Pre	43.0 (22.3)
	Post	− 4.3 (14.2)
	Δ	− 47.3 (22.6)
TK	Pre	23.0 (19.7)
	Post	36.5 (12.3)
	Δ	13.6 (15.5)
Fusion segment		9.2 (2.3)
LLIF segment		2.7 (0.8)
UIV (T1-T5/T6-T8/T9-12/L1-L2)		14/9/67/2
Operation time (min)	1^st^ (LLIF)	120.7 (50.6)
	2^nd^ (PSF)	331.0 (67.7)
	Total	450.4 (95.4)
Intraoperative EBL (ml)	1^st^ (LLIF)	113.3 (212.2)
	2^nd^ (PSF)	574.6 (465.6)
	Total	686.7 (500.8)

### Model development

Five supervised ML models were implemented to predict the occurrence of PJF after ASD surgery: Logistic Regression, SVM, Decision Tree, Naive Bayes, and Random Forest. All models were constructed using the scikit-learn library (v1.3.0, Python 3.10) with default parameters unless otherwise specified.

Five independent stratified trials were conducted using an 80:20 train-test split to evaluate model performance. In each trial, the test set consisted of 18 patients (14 non-PJF and 4 PJF), while the remaining 74 patients were used for training. The test sets across the five trials were designed to be mutually exclusive to prevent patient duplication and ensure independent evaluation. As the total number of patients was 92, this approach covered 90 unique patients across the five trials, with each evaluated exactly once. The remaining two patients were included exclusively in the training sets. This design maximized use of the available data while avoiding test set overlap and potential information leakage.

### Data preprocessing

Data Anonymization and Privacy: Patient data were anonymized to ensure privacy. For model development and analysis, sensitive information was protected in accordance with data protection standards, leveraging Python’s scikit-learn framework for secure processing while maintaining confidentiality.

Missing Data Handling: To enhance data quality and model performance, missing values were addressed by imputing missing data based on the median.

Feature Selection: A combined statistical and model-based feature selection strategy was employed to identify robust predictors of PJF. Initially, univariate t-tests were conducted to compare numerical variables between the PJF and non-PJF groups. In the initial univariate analysis, features with a *p*-value < 0.1 were considered potentially relevant for inclusion in the predictive model. Although this threshold does not meet the conventional standard for statistical significance, it was selected to allow the inclusion of clinically meaningful trends during exploratory analysis, particularly given the limited sample size and the multifactorial nature of PJF. Preoperative PI–LL and PT met this criterion and were thus retained for subsequent feature importance evaluation despite not achieving *p* < 0.05.

Subsequently, feature importance was evaluated using a random forest classifier trained on the entire dataset. The analysis highlighted several alignment-related parameters—Post PI–LL, Post TK, Pre TK, and Post LL—as top-ranking features. Additionally, Δ LL (change in lumbar lordosis) demonstrated significant group differences (*p* < 0.05) and contributed moderately to model performance, supporting its inclusion.

**Fig. 1:**
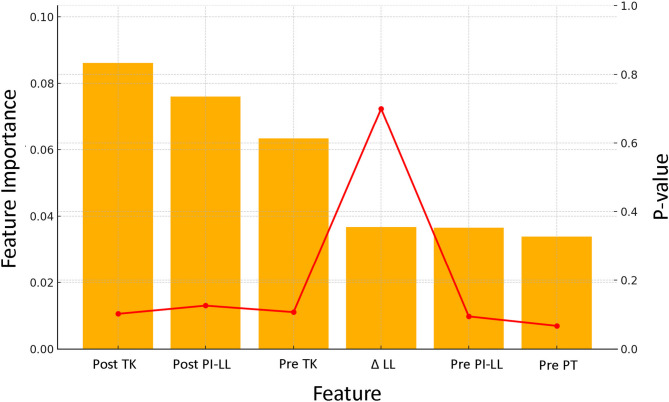
Feature selection for PJF prediction. Univariate t-tests identified preoperative PI–LL mismatch (Pre PI–LL) and pelvic tilt (Pre PT) as potential predictors (*p* < 0.1). Random forest feature importance highlighted postoperative PI–LL (Post PI–LL), thoracic kyphosis (Post TK, Pre TK), postoperative lumbar lordosis (Post LL), and change in lumbar lordosis (Diff LL) as key variables. Based on their predictive power and clinical relevance, six features—Post PI–LL, Post TK, Pre TK, Diff LL, Pre PI–LL, and Pre PT—were selected for model construction.

Based on both predictive value and clinical relevance, the following six features were selected for model construction (Fig. 1): Post PI–LL, Post TK, Pre TK, Diff LL (Δ LL), Pre PI–LL, and Pre PT. These variables capture both the preoperative spinal alignment and the corrective changes achieved postoperatively, reflecting current biomechanical understanding of PJF development. Notably, Pre PI–LL and Pre PT were retained in the model despite their borderline statistical significance due to their consistent contribution to prediction accuracy.

### Model training and tuning

Hyperparameter tuning for each model was conducted using grid search with stratified cross-validation to optimize performance. Feature importance was assessed to determine each variable’s contribution to predicting PJF. Specifically, stratified k-fold cross-validation (k = 5 and 10) was used to preserve the PJF/non-PJF class ratio within each fold, ensuring the model.

### Evaluation of machine learning models

In addition to the random forest model, four other ML algorithms—logistic regression, SVM, decision tree, and naive Bayes—were evaluated for their predictive performance in identifying PJF cases.

For logistic regression, coefficients were analyzed to understand the influence of each feature on PJF occurrence, with hyperparameter tuning for optimal regularization. The SVM model was evaluated with a radial basis function kernel to capture nonlinear relationships. Grid search was used to fine-tune C (penalty) and gamma values, enhancing the model’s ability to differentiate between PJF and non-PJF cases. For the decision tree model, maximum depth and minimum samples per split were adjusted to prevent overfitting. Feature importance scores were extracted to assess the influence of each variable on PJF prediction.　The naive Bayes model assumed a Gaussian distribution for continuous features and was selected for computational efficiency. Class priors were based on training data to align probability estimates with the observed distribution of PJF and non-PJF cases.

Each model was evaluated using fivefold cross-validation to estimate stability across metrics such as accuracy, AUC, precision, recall, and F1-score. Performance metrics were averaged across cross-validation folds to enable consistent comparison of model effectiveness. Although stratified sampling was used to balance PJF and non-PJF cases during cross-validation, other potential confounders (such as age, surgical technique, and severity of deformity) were not adjusted for in the modeling process.

### Random forest model implementation

The random forest classification was performed using Python’s scikit-learn library. For this analysis, the number of trees (n_estimators) was set to 500, and the number of features used at each split (max_features) was set to 5. This configuration was chosen to optimize the model’s Kappa value. The Random Forest model’s performance was specifically evaluated using fivefold and tenfold cross-validation to assess the robustness and consistency of its predictive accuracy for PJF. This method divided the data into five equal parts or “folds.” During each iteration, one-fold was held out as the validation set, while the remaining four were used for training the model. This process was repeated five times, with each fold serving as the validation set once. The model’s accuracy was recorded for each iteration, and the average accuracy across the five folds was calculated to provide a reliable estimate of its performance. This approach reduces the risk of overfitting and ensures that the model’s accuracy is robust across different subsets of the data.

### Evaluation metrics

The performance of each model was evaluated on the test set using the following metrics:

- *Receiver operating characteristic (ROC) analysis to calculate the Area Under the Curve (AUC)* The area under the ROC curve was calculated to assess the discriminative power of each model in identifying PJF cases.
- *Accuracy* Overall model correctness was calculated as the percentage of correct predictions.
- *Precision and recall* Precision and recall were calculated for each class (PJF and non-PJF cases) to evaluate the model’s performance in correctly identifying both PJF and non-cases.
- *F1-score* The F1-score was calculated to provide a balanced measure of precision and recall.

All analyses were conducted using Python’s scikit-learn library, and model performance metrics were reported to assess the robustness and clinical applicability of each PJF risk prediction model.

In addition to these conventional metrics, we also conducted a Decision Curve Analysis (DCA) to evaluate the clinical utility of the Random Forest model across a range of threshold probabilities (0.01 to 0.80). Net benefit was calculated at each threshold using the formula:

Net Benefit = (true positives (TP)/N) − (false positives (FP)/N) × (threshold/(1 − threshold)), where TP and FP denote the number of true and false positives, respectively, and N is the total number of patients. DCA enables comparison of the model’s net benefit against two extreme strategies: treating all patients as PJF-positive and treating none. This analysis was performed using the prediction outputs from the fifth independent test set to reflect realistic clinical use.

### Statistical analysis

Data are presented as mean ± standard deviation (SD) or patient count (%). The predictive performance of each model was evaluated using ROC analysis to calculate AUC values, with higher values indicating stronger discriminative power for distinguishing between PJF and non-PJF cases.

Predictive probabilities for PJF generated by the Random Forest model were further analyzed using the Mann–Whitney U test to compare PJF and non-PJF groups. Statistical significance was set at *p* < 0.05 to validate the model’s predictive capacity and clinical applicability.

## Results

To evaluate the consistency and predictive performance of ML algorithms for predicting PJF, five models—Logistic Regression, SVM, Decision Tree, Naive Bayes, and Random Forest—were tested across five independent stratified trials using six selected features.

**Fig. 2:**
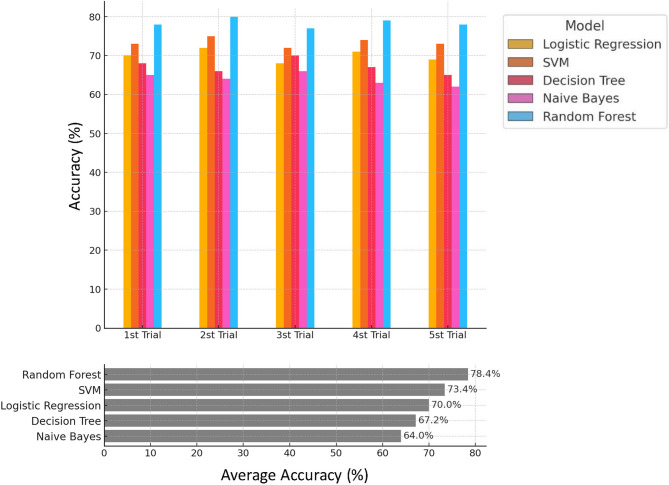
Model accuracy comparison across five trials. This figure illustrates the accuracy percentages of five machine learning models used to predict PJF across five separate trials. Each horizontal bar represents the average accuracy for a specific model—Logistic Regression, Support Vector Machine, Decision Tree, Naive Bayes, and Random Forest—in each trial, labeled as “1st Trial” through “5th Trial.” Accuracy is displayed as a percentage, with higher values indicating better predictive performance. The trials demonstrate the models’ consistency and variation in accuracy across repeated sampling. The lower figure shows the average accuracy across five trials.

The upper panel of Fig. 2 presents the accuracy scores for each model across the five trials. While moderate variability was observed between trials, the Random Forest and SVM models consistently demonstrated superior accuracy compared to the others. For example, Random Forest achieved accuracies of 80.0% and 79.0% in Trials 2 and 4, respectively, whereas SVM maintained stable performance around 73–75% across all trials. The lower panel of Fig. 2 summarizes the average accuracy across the five trials. The Random Forest model achieved the highest mean accuracy at 78.4%, followed by SVM (73.4%) and Logistic Regression (70.0%). In contrast, Naive Bayes and Decision Tree models showed lower performance, with mean accuracies of 64.0% and 67.2%, respectively.

These results suggest that Random Forest, due to its ensemble-based architecture and robustness to noise, provides the most reliable predictive performance for PJF among the models tested. SVM also showed competitive and stable performance, making it a viable secondary option, particularly when model interpretability is not a primary concern.

**Fig. 3:**
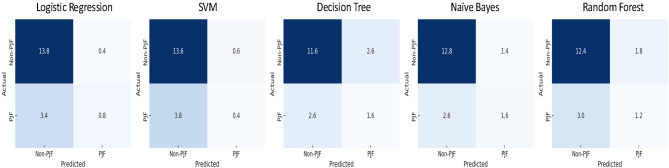
Confusion matrices for each model averaged over five trials. This figure presents the average confusion matrices for five machine learning models—Logistic regression, support vector machine, decision tree, naive bayes, and random forest—used to predict PJF in spinal surgery patients. These averaged confusion matrices provide insight into the models’ predictive accuracy and error distribution, highlighting their strengths and limitations in classifying PJF presence and absence.

To further assess classification performance, confusion matrices were generated and averaged across the five stratified trials (Fig. 3). These matrices provide detailed insights into each model’s distribution of TP, true negatives (TN), FP, and false negatives (FN).

Among all models, Random Forest showed the best overall balance, with an average TP of 1.2 and a relatively low FP (1.8), indicating consistent yet conservative identification of PJF cases. Naive Bayes exhibited higher sensitivity with the highest TP (1.6) but also showed a slightly elevated FP rate (1.4), suggesting a trade-off between sensitivity and specificity. Decision Tree matched Naive Bayes in TP (1.6) but recorded the highest FP (2.6), reflecting a tendency to overpredict PJF. In contrast, Logistic Regression and SVM demonstrated limited capability in identifying PJF cases, with average TP values of 0.8 and 0.4, respectively, and relatively high FN counts.

**Fig. 4:**
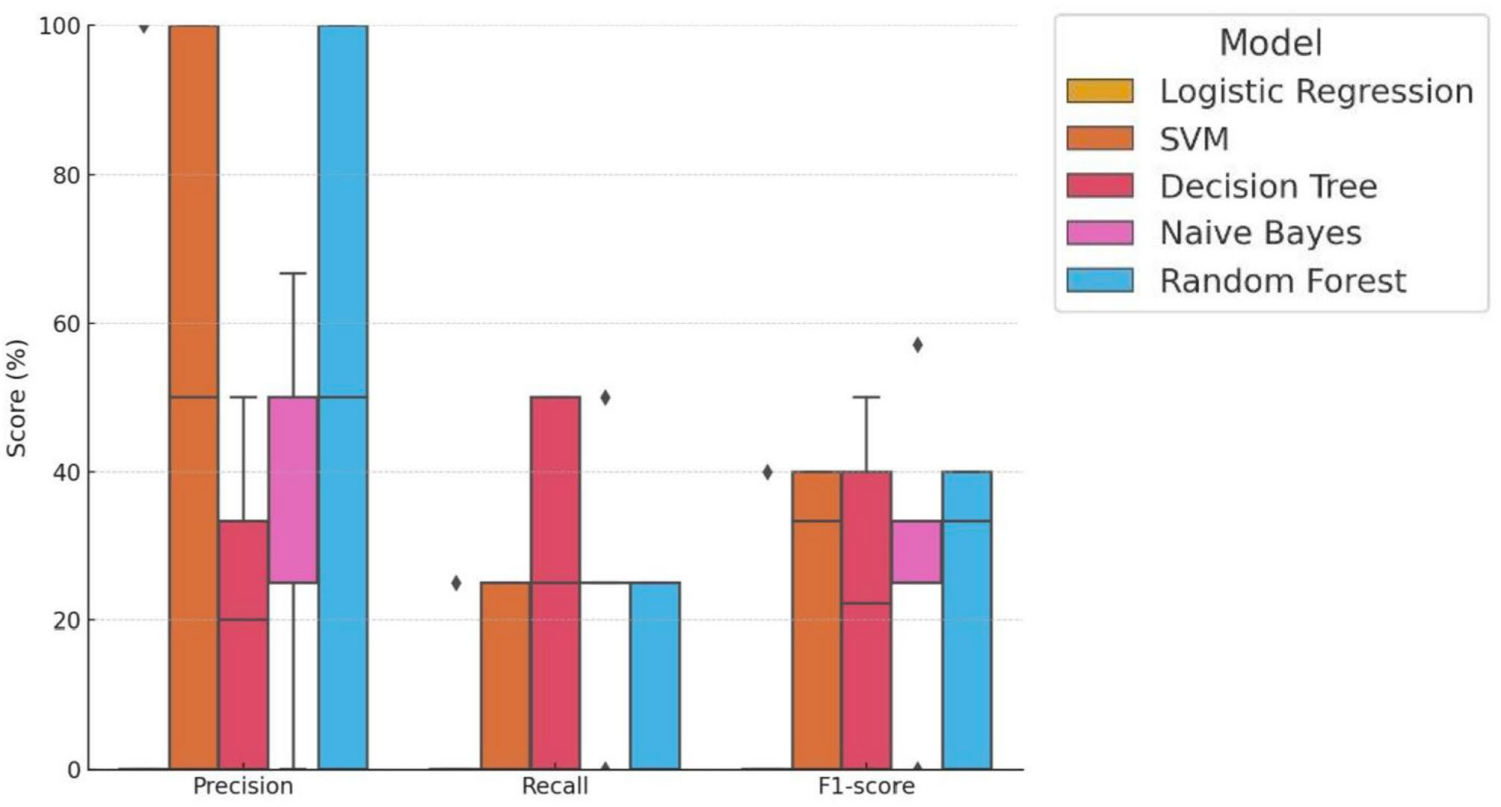
Distribution of precision, recall, and f1-score across five trials. Box plots illustrate the distribution of precision, recall, and F1-score for each machine learning model based on five independent trials. Metrics are shown as percentages. Random Forest and Naive Bayes demonstrated consistently higher and more stable performance in both recall and F1-score, suggesting strong capability in identifying PJF cases.

In addition, model-wise performance metrics—precision, recall, and F1-score—were calculated across the five trials and visualized using box plots (Fig. 4).

Random Forest achieved a median F1-score of 33.3%, indicating a balanced performance between precision and recall, despite some variability across trials. The model’s average F1-score was 22.7%, with at least one trial exhibiting notably low performance, which lowered the overall mean. This modest F1-score primarily reflects the class imbalance in the dataset, as only 21 of 92 patients (22.8%) experienced PJF, making it challenging to achieve high precision and recall simultaneously. Naive Bayes followed with a median F1-score of 25.0% and a slightly higher mean of 28.1%, suggesting relatively greater consistency. Decision Tree also demonstrated competitive but more variable performance (median: 22.2%, mean: 22.4%). SVM yielded a median F1-score of 33.3%, comparable to Random Forest, but with a lower mean (22.7%). Logistic Regression had the lowest performance, with a median F1-score of 0.0% and a mean of 8.0%, indicating a poor ability to identify PJF cases.

**Fig. 5:**
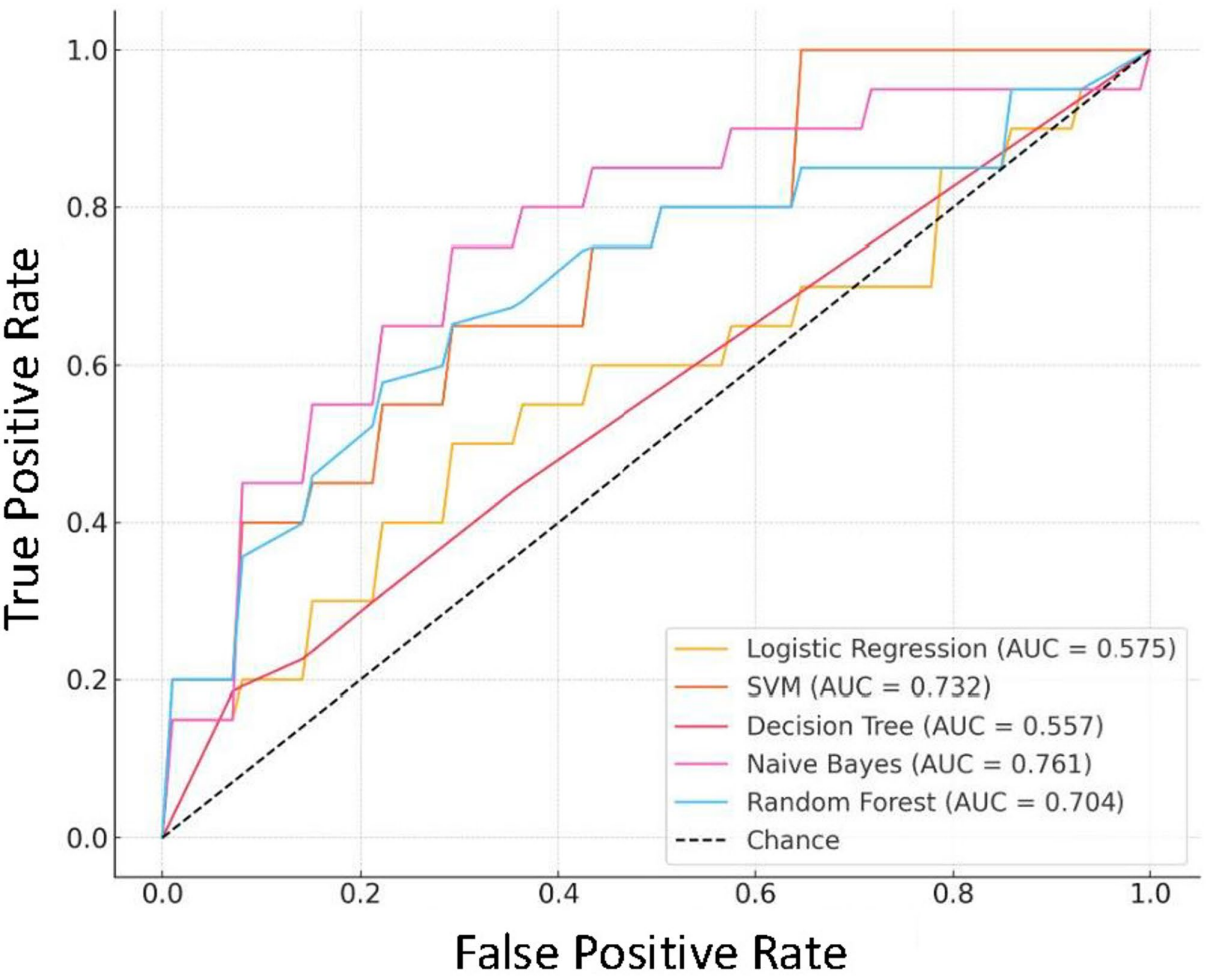
Average ROC Curves for PJF Prediction Using Five Models Across Five Trials. This figure illustrates the average Receiver Operating Characteristic (ROC) curves for five machine learning models used to predict PJF. The ROC curves represent the trade-off between sensitivity (true positive rate) and specificity (1—false positive rate) for each model across five trials, providing an overall view of each model’s discriminative power.

To further examine model discrimination, average ROC curves were generated for each machine learning model across the five trials (Fig. 5). The ROC curves illustrate the trade-off between sensitivity and specificity, offering a comprehensive view of each model’s classification capability. Among the five models, Naive Bayes achieved the highest average area under the curve (AUC = 0.761), followed by SVM (AUC = 0.732) and Random Forest (AUC = 0.704), all indicating moderate-to-good discriminative power. In contrast, Logistic Regression (AUC = 0.575) and Decision Tree (AUC = 0.557) showed limited discrimination, likely due to the class imbalance and complex nonlinear relationships among features.

**Fig. 6:**
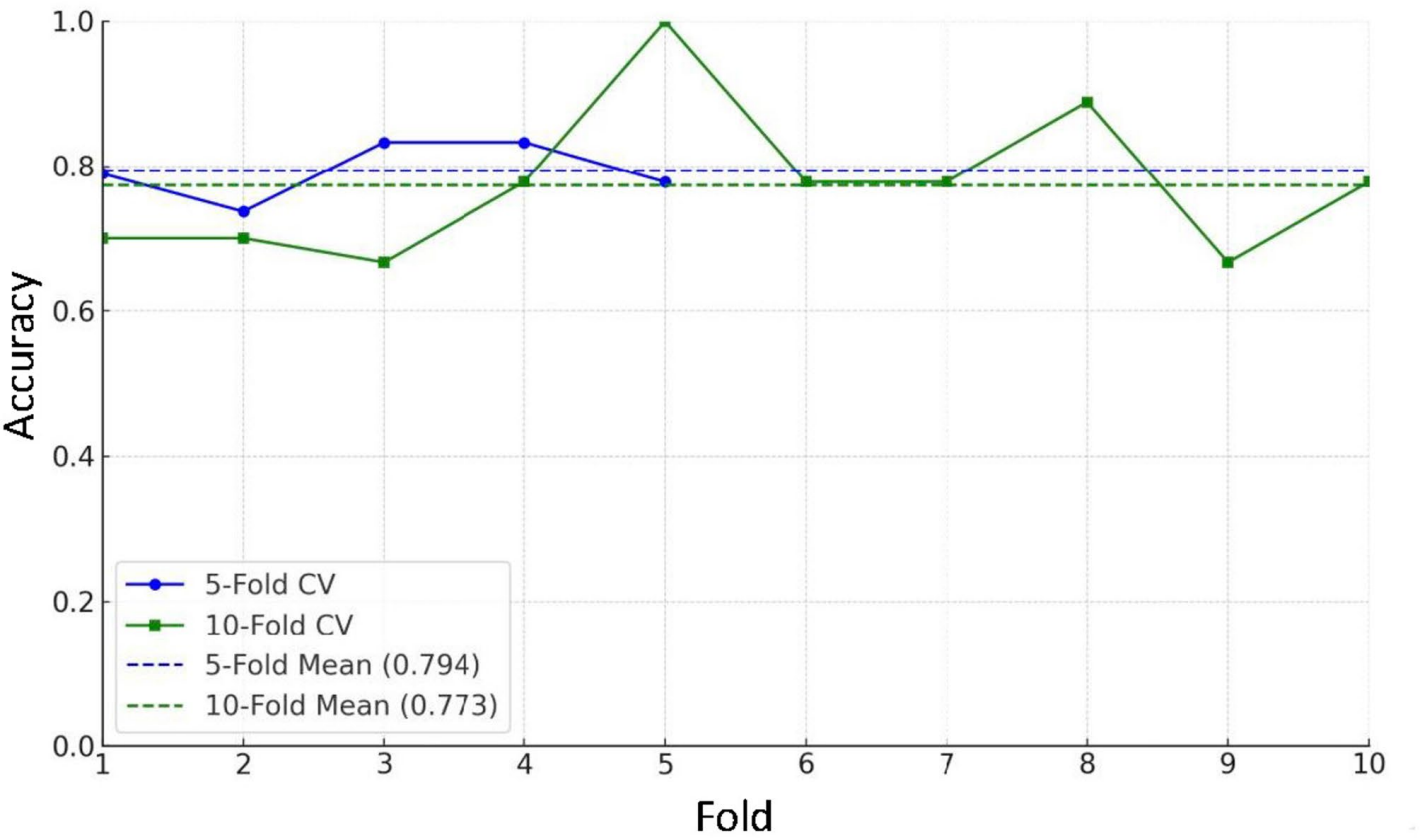
Comparison of 5-Fold and 10-Fold Cross-Validation Accuracy Scores for PJF Prediction Model. This figure compares the accuracy scores obtained from fivefold and tenfold cross-validation using a Random Forest model to predict PJF in adult spinal deformity surgery patients. The blue line with circles represents the accuracy scores across five folds, while the green line with squares represents the scores across ten folds. Dashed lines indicate the mean accuracy for each cross-validation method: fivefold (78.1%) and tenfold (77.1%). The y-axis range is set from 0 to 1 for clear visualization.

Figure 6 compares the accuracy scores of the Random Forest model using fivefold and tenfold stratified cross-validation. The fivefold cross-validation resulted in a slightly higher average accuracy (79.4%) than the tenfold cross-validation (77.3%). Minor variability in accuracy was observed across folds in both methods. The consistency across folds suggests that the Random Forest model maintains stable predictive performance regardless of the validation scheme. However, the marginally higher mean accuracy in the fivefold setup indicates that this approach may be more suitable for this dataset and task.

**Fig. 7:**
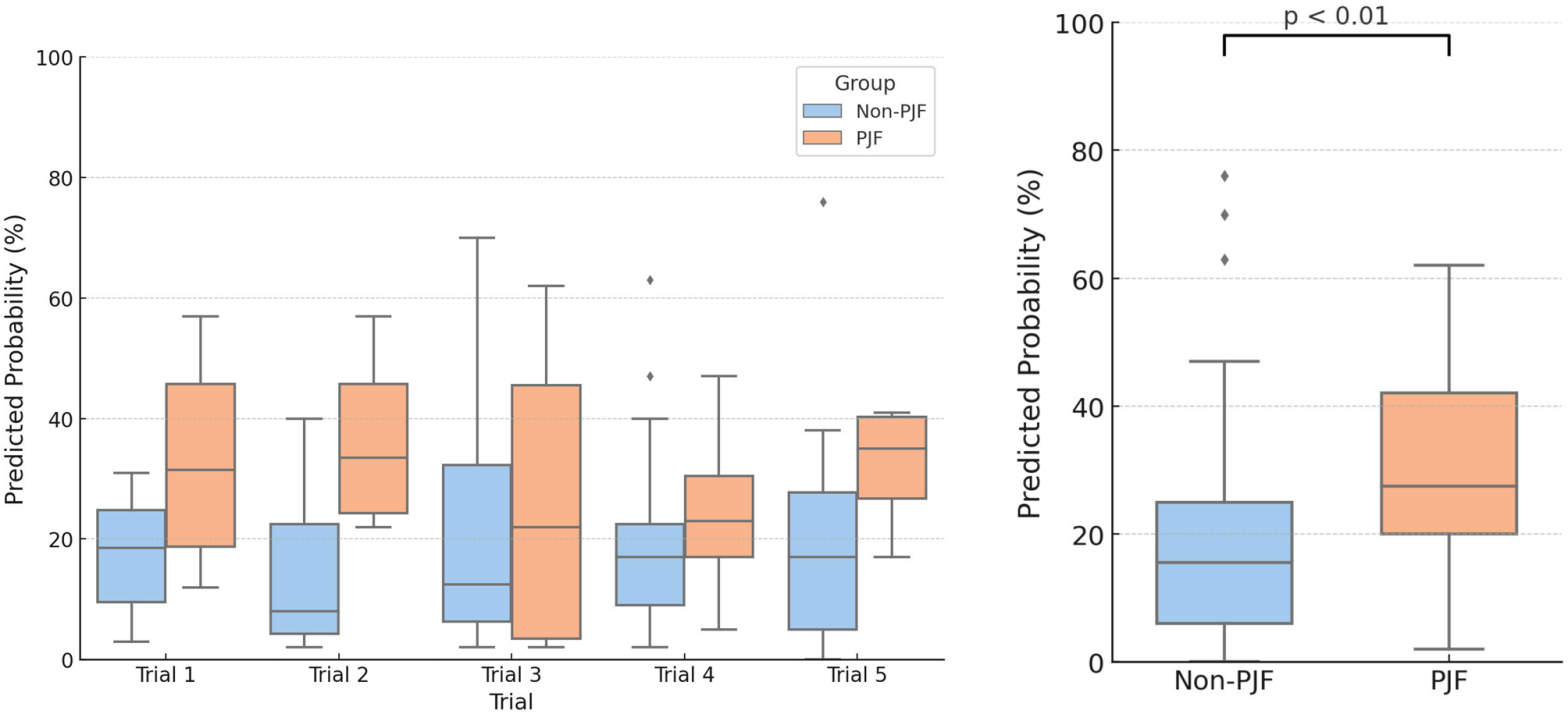
Predicted Probability of PJF in PJF and Non-PJF Groups Averaged Over Five Trials Using Random Forest. Upper panel: Box plots show the distribution of predicted probabilities for proximal junctional failure (PJF) as generated by the Random Forest model across five independent test trials (Trial 1–5). In each trial, 18 patients (including 4 PJF cases and 14 non-PJF cases) were independently sampled. The model consistently assigned higher predicted probabilities to PJF cases than to non-PJF cases. Lower panel: Combined box plots compare the predicted probabilities between PJF and non-PJF groups across all test samples aggregated from five trials (n = 90). The median predicted probability was higher in the PJF group (27.5%) than in the non-PJF group (15.5%), and this difference was statistically significant (*p* < 0.01, Mann–Whitney U test).

To further explore the discriminative power of the Random Forest model, predicted probabilities of PJF were analyzed across five independent test trials (Fig. 7). The PJF group consistently exhibited higher predicted probabilities than the non-PJF group in every trial. When pooling results across trials, the average predicted probability for the PJF group was significantly higher than that for the non-PJF group (mean ± SD: 30.6 ± 18.1% vs. 18.6 ± 16.4%). This difference was statistically significant (*p* = 0.0057, Mann–Whitney U test), highlighting the model’s capacity to distinguish between patients who developed PJF and those who did not.

To further evaluate the clinical utility of the Random Forest model, we performed DCA using the prediction probabilities from the fifth independent test set. The DCA curve showed that the model provided a higher net benefit compared to both the “treat-all” and “treat-none” strategies across a clinically relevant range of threshold probabilities (0.10 to 0.40). This indicates that model-guided intervention may improve clinical decision-making by identifying high-risk patients for PJF while minimizing unnecessary treatment in low-risk individuals (Supplemental Table 1).

Table 2 presents representative test data from the Random Forest model. The predicted probabilities for the PJF group were [0.62, 0.54, 0.77, 0.61], with a mean ± SD of 0.635 ± 0.097. In contrast, the non-PJF group had probabilities of [0.00, 0.02, 0.18, 0.00, 0.10, 0.06, 0.10, 0.13, 0.05, 0.03, 0.04, 0.13, 0.00, 0.00], with a mean ± SD of 0.060 ± 0.059.

**Table 2:** Test data patient Demographics, Spinal Alignment Parameters, and Changes Following Spinal Fusion Surgery for Adult Spinal Deformity. (A) Patient Demographics and Preoperative Spinal Alignment Parameters. This table overviews age, sex, PJF status, preoperative alignment measurements (SVA, PI, PT, SS, LL, PI-LL, TK), and PJF prediction probability for each patient. (B) Postoperative Spinal Alignment Parameters. This table presents postoperative alignment measurements (SVA, PI, PT, SS, LL, PI-LL, TK) for each patient following spinal fusion surgery. (C) Changes in Spinal Alignment Parameters (Δ) from Preoperative to Postoperative. This table shows the changes in alignment parameters (ΔSVA, ΔPI, ΔPT, ΔSS, ΔLL, ΔPI-LL, ΔTK), indicating the surgical impact on each parameter for each patient.

(A)										
No	Age	Sex	PJF	Pre	Pre	Pre	Pre	Pre	Pre	Pre
				SVA	PI	PT	SS	LL	PI-LL	TK
1	77	F	No	158.4	53.7	38.2	15.5	− 4.6	58.3	16.0
2	73	F	No	187.2	58.0	42.3	15.7	0.3	57.7	21.8
3	61	F	Yes	211.1	43.3	30.5	12.8	− 23.7	67.0	− 15.3
4	70	F	No	98.1	42.8	36.1	6.7	25.6	17.2	58.8
5	72	F	Yes	389.7	57.4	40.3	17.1	− 32.4	89.8	21.5
6	54	F	Yes	36.6	33.0	17.1	15.9	46.9	− 13.9	79.4
7	69	F	No	207.0	53.0	28.6	24.4	− 1.8	54.8	3.6
8	56	F	No	96.1	40.6	19.2	21.4	0.7	39.9	10.8
9	81	F	No	173.7	56.6	47.6	9.0	− 6.7	63.3	16.0
10	81	F	Yes	177.0	41.6	26.2	15.4	2.2	39.4	40.1
11	68	F	No	206.3	51.4	37.3	14.1	0.6	50.8	30.9
12	71	F	No	207.4	49.0	39.5	9.5	17.3	31.7	14.8
13	67	F	No	295.0	53.2	39.7	13.5	− 37.5	90.7	5.1
14	69	F	No	174.6	32.0	53.4	-21.4	− 51.1	83.1	0.1
15	81	M	No	225.1	54.9	38.6	16.3	0.6	54.3	33.6
16	73	F	No	208.8	52.0	43.2	8.8	− 22.2	74.2	10.4
17	78	F	No	205.8	58.3	47.2	11.1	− 18.9	77.2	− 1.7
18	80	F	No	93.6	49.4	24.7	24.7	40.6	8.8	45.0

(B)							
No	Post	Post	Post	Post	Post	Post	Post
	SVA	PI	PT	SS	LL	PI-LL	TK
1	16.9	44.0	10.4	33.6	52.1	− 8.1	24.3
2	5.3	53.4	23.1	30.3	48.3	5.1	29.4
3	139.6	32.5	21.9	10.6	11.8	20.7	13.0
4	19.3	44.0	19.0	25.0	40.5	3.5	42.9
5	− 1.1	51.0	11.1	39.9	56.3	− 5.3	23.8
6	− 10.6	34.3	− 3.6	37.9	64.4	− 30.1	59.7
7	48.5	51.7	13.8	37.9	48.8	2.9	34.5
8	35.9	32.2	3.5	28.7	57.0	− 24.8	27.0
9	− 19.8	48.6	20.1	28.5	42.7	5.9	27.5
10	21.1	38.1	− 12.4	50.5	79.3	− 41.2	57.6
11	− 31.1	46.9	18.0	28.9	66.9	− 20.0	56.3
12	− 57.1	50.7	22.6	28.1	62.4	− 11.7	31.9
13	49.1	48.7	7.6	41.1	51.5	− 2.8	17.3
14	21.2	30.6	14.6	16.0	24.1	6.5	27.2
15	132.4	54.1	22.2	31.9	34.4	19.7	39.7
16	75.9	42.5	11.7	30.8	33.9	8.6	20.5
17	32.9	53.9	24.5	29.4	45.2	8.7	37.0
18	77.8	52.2	26.6	25.6	40.6	11.6	39.2

(C)								
No	Δ SVA	Δ PI	Δ PT	Δ SS	Δ LL	Δ PI-LL	Δ TK	PJF Prediction probability
1	− 141.5	− 9.7	− 27.8	18.1	56.7	− 66.4	8.3	0.04
2	− 181.9	− 4.6	− 19.2	14.6	48.0	− 52.6	7.6	0.02
3	− 71.5	− 10.8	− 8.6	− 2.2	35.5	− 46.3	28.3	0.54
4	− 78.8	1.2	− 17.1	18.3	14.9	− 13.7	− 15.9	0.03
5	− 390.8	− 6.4	− 29.2	22.8	88.7	− 95.1	2.3	0.61
6	− 47.2	1.3	− 20.7	22.0	17.5	− 16.2	− 19.7	0.77
7	− 158.5	− 1.3	− 14.8	13.5	50.6	− 51.9	30.9	0.05
8	− 60.2	− 8.4	− 15.7	7.3	56.3	− 64.7	16.2	0.13
9	− 193.5	− 8.0	− 27.5	19.5	49.4	− 57.4	11.5	0
10	− 155.9	− 3.5	− 38.6	35.1	77.1	− 80.6	17.5	0.62
11	− 237.4	− 4.5	− 19.3	14.8	66.3	− 70.8	25.4	0.13
12	− 264.5	1.7	− 16.9	18.6	45.1	− 43.4	17.1	0.06
13	− 245.9	− 4.5	− 32.1	27.6	89.0	− 93.5	12.2	0.18
14	− 153.4	− 1.4	− 38.8	37.4	75.2	− 76.6	27.1	0
15	− 92.7	− 0.8	− 16.4	15.6	33.8	− 34.6	6.1	0.1
16	− 132.9	− 9.5	− 31.5	22.0	56.1	− 65.6	10.1	0
17	− 172.9	− 4.4	− 22.7	18.3	64.1	− 68.5	38.7	0
18	− 15.8	2.8	1.9	0.9	0	2.8	− 5.8	0.1

**Fig. 8:**
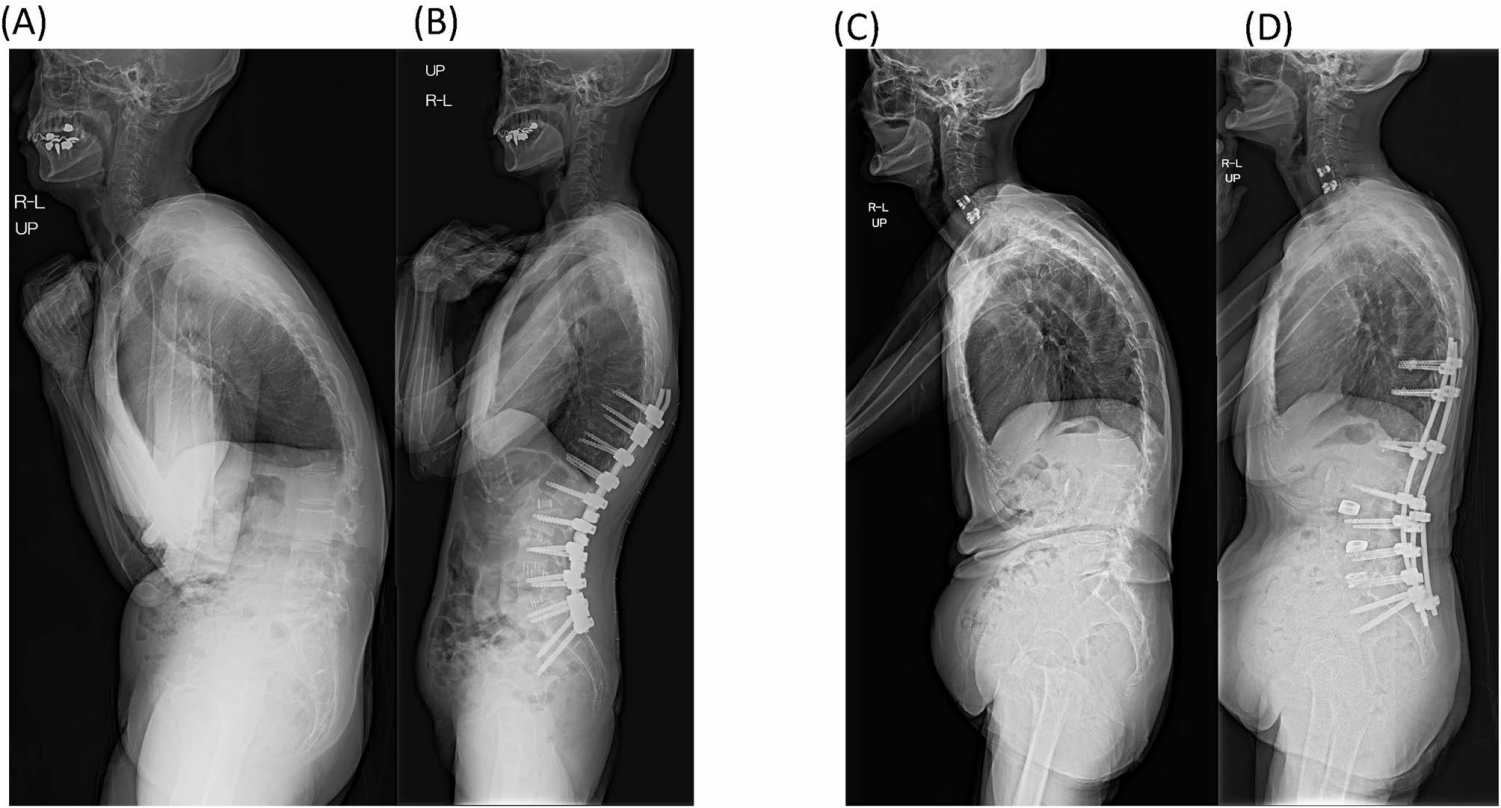
Representative Radiographs from PJF and Non-PJF Cases. This figure shows preoperative and postoperative standing lateral radiographs for one patient who developed PJF and one who did not. (**A**) Preoperative radiograph of a patient who developed PJF. (**B**) Postoperative radiograph of the same patient. (**C**) Preoperative radiograph of a patient who did not develop PJF. (**D**) Postoperative radiograph of the same patient.

Figure 8 presents preoperative and postoperative standing lateral radiographs from two representative cases in the current study cohort—one patient who developed PJF and one who did not.

## Discussion

This study evaluated the performance of five ML models in predicting PJF among patients with ASD undergoing corrective surgery, focusing on spinal alignment parameters and their surgical changes. Among the tested models, the Random Forest algorithm achieved the highest mean accuracy and AUC, demonstrating its effectiveness in handling the nonlinear and multifactorial factors associated with PJF. In clinical decision-making, artificial intelligence and ML are increasingly being recognized as powerful tools in spine surgery, particularly for predicting outcomes of operative and nonoperative management^21^.

In contrast, conventional models such as logistic regression and SVM showed limited ability to identify true PJF cases, as reflected by their low recall and AUC values. Naive Bayes and decision tree models demonstrated moderate sensitivity but greater variability across trials. These findings highlight the advantages of ensemble learning methods like Random Forest in capturing complex biomechanical interactions inherent to spinal surgery outcomes.

A key strength of our approach lies in the incorporation of both preoperative and postoperative alignment parameters, along with their changes (Δ), thereby reflecting the extent of surgical correction. Based on these dynamic variables, the Random Forest model effectively distinguished between patients with and without PJF. The model’s predicted probabilities were significantly higher in the PJF group (mean ± SD: 30.6 ± 18.1%) compared to the non-PJF group (18.6 ± 16.4%, *p* = 0.0057), underscoring its potential clinical utility.

These results emphasize the importance of selecting appropriate models for optimal predictive performance. Our findings suggest that more complex models like Random Forest are better suited to capture the nonlinear relationships involved in PJF risk. To supplement conventional metrics and better assess real-world utility, we conducted DCA. The DCA indicated that across threshold probabilities of approximately 0.1 to 0.4, the Random Forest model demonstrated a higher net benefit than the “treat all” and “treat none” strategies. This suggests the model can provide meaningful guidance in identifying patients who may benefit from enhanced postoperative surveillance or targeted interventions, supporting its application in real-world clinical decision-making. Recent work employing a modified Global Alignment and Proportion score, combined with body mass index and bone mineral density, has similarly shown the utility of Random Forest models in predicting mechanical complications after ASD surgery^22^. As in our study, incorporating comprehensive patient factors into machine learning frameworks significantly improved predictive accuracy. Several other studies have applied deep learning algorithms, including Random Forest and neural networks, to spine-related prediction tasks^23–25^, highlighting the promise of advanced AI methods in addressing the multifactorial nature of spinal complications. Notably, our model was developed using preoperative and early postoperative alignment parameters, whereas the occurrence of PJF defined the outcomes during the entire follow-up period (≥ 1 year). Therefore, the predictive analysis is not limited to 1-year outcomes but captures both early- and late-onset PJF cases.

This study is one of the first to assess PJF risk following ASD surgery comparatively using multiple ML models. Despite encouraging results, several limitations must be acknowledged.

First, this study was retrospective in nature, which may introduce bias in data collection and patient selection, potentially limiting the generalizability of the findings. Although the sample size of 92 patients is reasonable for conducting an exploratory analysis, it remains limited for ensuring the robustness and broad applicability of the predictive model. As such, the cohort may not fully capture the clinical diversity observed in broader populations. Furthermore, all patients were East Asian individuals treated at a single academic institution in Japan, resulting in restricted racial and geographic diversity. Therefore, external validation in larger, multicenter, and multi-ethnic cohorts is essential.

Second, the limited sample size raises concerns about potential model overfitting. To mitigate this risk, we performed both fivefold and tenfold stratified cross-validation, which demonstrated consistent accuracy values. Stratified splitting ensured a balanced representation of PJF and non-PJF cases in each fold; nevertheless, independent validation using external datasets remains necessary.

Third, although the model incorporated key demographic and radiographic features, several clinically relevant variables—such as bone mineral density, comorbidity profiles (e.g., diabetes, osteoporosis), and frailty metrics—were not consistently available in our retrospective dataset and were therefore excluded. For example, CT-based Hounsfield Unit measurements were available for only 79.3% of patients, and standardized assessments such as the Charlson Comorbidity Index or Modified Frailty Index were not routinely collected. Given the known influence of bone quality and systemic health on postoperative mechanical stability, omitting these factors may have reduced the model’s predictive accuracy. We recognize that their inclusion in future prospective, multicenter studies could enhance both predictive performance and clinical applicability.

Fourth, surgical technique parameters—such as osteotomy type, rod material, upper instrumented vertebra selection, rod contouring methods, and screw placement techniques—were not included in the present model. While the overall surgical strategy in this cohort was standardized (two-stage LLIF followed by posterior spinal fusion), these intraoperative variations can influence construct rigidity, junctional stress distribution, and ultimately, the risk of PJF. Future studies should systematically capture and analyze surgical technique details alongside patient-specific factors to improve the comprehensiveness of predictive models.

Fifth, although stratified cross-validation was applied, no formal subgroup analyses or adjustments for potential confounders (e.g., age, deformity severity, surgical method) were performed. Residual confounding may therefore have influenced model performance. Future work should explore stratified modeling approaches that account for these variables, potentially enabling risk prediction tailored to specific patient subgroups.

Sixth, sagittal and coronal deformities were not analyzed as independent predictors. Each deformity plane may carry distinct biomechanical implications and predictive relevance; thus, incorporating alignment changes in both planes could further improve model performance and clinical applicability.

Despite these limitations, the Random Forest model showed substantial promise for predicting PJF risk. Although predictive power was modest due to class imbalance, the model represents a valuable step toward risk stratification in clinical practice. By integrating alignment parameters with comprehensive patient-specific data—including bone quality, comorbidities, frailty status, muscle mass, and detailed surgical technique characteristics—and validating the model in diverse, multicenter cohorts, it may be possible to develop robust, individualized prediction tools. Such tools could assist surgeons in optimizing preoperative planning, tailoring intraoperative strategies, and implementing targeted postoperative surveillance protocols to reduce the incidence of PJF.

## Conclusions

This study demonstrates the feasibility and predictive performance of ML models—notably the Random Forest algorithm—in identifying patients at risk of PJF following ASD surgery. Among the models evaluated, Random Forest consistently outperformed the others regarding accuracy, F1-score, and AUC, and effectively distinguished between PJF and non-PJF cases using preoperative and postoperative alignment parameters.

These findings support the integration of data-driven predictive tools into preoperative planning to enable personalized risk stratification, optimize surgical decision-making, and potentially reduce the incidence of PJF. External validation in diverse populations and incorporating additional clinical variables—such as comorbidities and bone mineral density—will enhance the model’s generalizability and clinical applicability.

## Supplementary Information

Below is the link to the electronic supplementary material.

Supplementary Material 1

## Data Availability

The data supporting the findings of this study are not publicly available due to patient confidentiality and institutional regulations. Therefore, the dataset cannot be shared externally. However, summary statistics and aggregated results can be provided upon reasonable request by contacting the corresponding author at a.hiyama@tokai.ac.jp.
