# Supplementary material for: Predicting proximal junctional failure in adult spinal deformity patients using machine learning models based on spinal alignment parameters
Source: Sci Rep. 2025 Nov 20;15:41127. doi: 10.1038/s41598-025-24872-1 (PMC12635256; doi:10.1038/s41598-025-24872-1)
Supplement: Supplementary file 1 — Supplementary Material 1 [file 41598_2025_24872_MOESM1_ESM.docx]

Supplemental Table 1.

Decision Curve Analysis (DCA) results across varying threshold probabilities for predicting proximal junctional failure (PJF) using a Random Forest model.

This table presents the net benefit values calculated at threshold probabilities ranging from 0.01 to 0.99 (in 0.01 increments). Net benefit quantifies the clinical utility of the model in comparison to two default strategies: treating all patients as high-risk for PJF ("Treat All") versus treating none ("Treat None").

Threshold Probability: The probability cut-off above which a patient is classified as high-risk for PJF.

Net Benefit (Model): The clinical benefit of using the Random Forest model at each threshold.

Net Benefit (Treat All): The benefit if all patients were considered high-risk and treated.

Net Benefit (Treat None): The baseline scenario in which no patient receives treatment (set to zero).

| Threshold Probability | Net Benefit  (Model) | Net Benefit  (Treat All) | Net Benefit  (Treat None) |
| --- | --- | --- | --- |
| 0.01 | 0.222 | 0.220 | 0 |
| 0.02 | 0.217 | 0.213 | 0 |
| 0.03 | 0.213 | 0.204 | 0 |
| 0.04 | 0.209 | 0.196 | 0 |
| 0.05 | 0.205 | 0.188 | 0 |
| 0.06 | 0.205 | 0.179 | 0 |
| 0.07 | 0.205 | 0.170 | 0 |
| 0.08 | 0.204 | 0.161 | 0 |
| 0.09 | 0.205 | 0.152 | 0 |
| 0.1 | 0.203 | 0.143 | 0 |
| 0.11 | 0.203 | 0.133 | 0 |
| 0.12 | 0.203 | 0.123 | 0 |
| 0.13 | 0.202 | 0.113 | 0 |
| 0.14 | 0.205 | 0.103 | 0 |
| 0.15 | 0.209 | 0.092 | 0 |
| 0.16 | 0.210 | 0.081 | 0 |
| 0.17 | 0.208 | 0.070 | 0 |
| 0.18 | 0.207 | 0.059 | 0 |
| 0.19 | 0.205 | 0.047 | 0 |
| 0.2 | 0.207 | 0.035 | 0 |
| 0.21 | 0.211 | 0.023 | 0 |
| 0.22 | 0.210 | 0.011 | 0 |
| 0.23 | 0.212 | -0.002 | 0 |
| 0.24 | 0.215 | -0.015 | 0 |
| 0.25 | 0.214 | -0.029 | 0 |
| 0.26 | 0.213 | -0.043 | 0 |
| 0.27 | 0.220 | -0.057 | 0 |
| 0.28 | 0.224 | -0.072 | 0 |
| 0.29 | 0.228 | -0.087 | 0 |
| 0.3 | 0.228 | -0.102 | 0 |
| 0.31 | 0.228 | -0.118 | 0 |
| 0.32 | 0.228 | -0.135 | 0 |
| 0.33 | 0.228 | -0.152 | 0 |
| 0.34 | 0.228 | -0.169 | 0 |
| 0.35 | 0.228 | -0.187 | 0 |
| 0.36 | 0.228 | -0.206 | 0 |
| 0.37 | 0.228 | -0.225 | 0 |
| 0.38 | 0.228 | -0.245 | 0 |
| 0.39 | 0.228 | -0.265 | 0 |
| 0.4 | 0.228 | -0.286 | 0 |
| 0.41 | 0.228 | -0.308 | 0 |
| 0.42 | 0.228 | -0.331 | 0 |
| 0.43 | 0.228 | -0.354 | 0 |
| 0.44 | 0.228 | -0.378 | 0 |
| 0.45 | 0.228 | -0.403 | 0 |
| 0.46 | 0.228 | -0.429 | 0 |
| 0.47 | 0.228 | -0.456 | 0 |
| 0.48 | 0.228 | -0.484 | 0 |
| 0.49 | 0.228 | -0.513 | 0 |
| 0.5 | 0.228 | -0.543 | 0 |
| 0.51 | 0.228 | -0.575 | 0 |
| 0.52 | 0.228 | -0.608 | 0 |
| 0.53 | 0.228 | -0.642 | 0 |
| 0.54 | 0.228 | -0.678 | 0 |
| 0.55 | 0.228 | -0.715 | 0 |
| 0.56 | 0.228 | -0.754 | 0 |
| 0.57 | 0.228 | -0.795 | 0 |
| 0.58 | 0.228 | -0.837 | 0 |
| 0.59 | 0.228 | -0.882 | 0 |
| 0.6 | 0.228 | -0.929 | 0 |
| 0.61 | 0.228 | -0.979 | 0 |
| 0.62 | 0.217 | -1.031 | 0 |
| 0.63 | 0.207 | -1.086 | 0 |
| 0.64 | 0.196 | -1.144 | 0 |
| 0.65 | 0.196 | -1.205 | 0 |
| 0.66 | 0.185 | -1.270 | 0 |
| 0.67 | 0.174 | -1.339 | 0 |
| 0.68 | 0.163 | -1.412 | 0 |
| 0.69 | 0.152 | -1.489 | 0 |
| 0.7 | 0.141 | -1.572 | 0 |
| 0.71 | 0.130 | -1.661 | 0 |
| 0.72 | 0.130 | -1.756 | 0 |
| 0.73 | 0.120 | -1.858 | 0 |
| 0.74 | 0.109 | -1.968 | 0 |
| 0.75 | 0.098 | -2.087 | 0 |
| 0.76 | 0.087 | -2.216 | 0 |
| 0.77 | 0.076 | -2.355 | 0 |
| 0.78 | 0.076 | -2.508 | 0 |
| 0.79 | 0.076 | -2.675 | 0 |
| 0.8 | 0.065 | -2.859 | 0 |
| 0.81 | 0.065 | -3.062 | 0 |
| 0.82 | 0.065 | -3.287 | 0 |
| 0.83 | 0.065 | -3.540 | 0 |
| 0.84 | 0.065 | -3.823 | 0 |
| 0.85 | 0.054 | -4.145 | 0 |
| 0.86 | 0.054 | -4.512 | 0 |
| 0.87 | 0.054 | -4.936 | 0 |
| 0.88 | 0.043 | -5.431 | 0 |
| 0.89 | 0.022 | -6.016 | 0 |
| 0.9 | 0.022 | -6.717 | 0 |
| 0.91 | 0.011 | -7.575 | 0 |
| 0.92 | 0.011 | -8.647 | 0 |
| 0.93 | 0.000 | -10.025 | 0 |
| 0.94 | 0.000 | -11.862 | 0 |
| 0.95 | 0.000 | -14.435 | 0 |
| 0.96 | 0.000 | -18.293 | 0 |
| 0.97 | 0.000 | -24.725 | 0 |
| 0.98 | 0.000 | -37.587 | 0 |
| 0.99 | 0.000 | -76.174 | 0 |
